# Uveal melanoma in the Iranian population: two decades of patient management in a tertiary eye center

**DOI:** 10.1186/s40942-024-00531-x

**Published:** 2024-03-01

**Authors:** Hamid Riazi-Esfahani, Abdulrahim Amini, Babak Masoomian, Mehdi Yaseri, Siamak Sabour, Ali Rashidinia, Mojtaba Arjmand, Seyed Mohsen Rafizadeh, Mohammadkarim Johari, Elias Khalili Pour, Fariba Ghassemi

**Affiliations:** 1 Ocular Oncology Service, Farabi Eye Hospital, Tehran University of Medical Sciences, South Kargar Street, Qazvin Square, Tehran, Iran; 2 Retina Service, Farabi Eye Hospital, Tehran University of Medical Sciences, Tehran, Iran; 3 Department of Ophthalmology, School of Medicine, Hormozgan University of medical sciences, Bandar Abbas, Iran; 4 Department of Epidemiology and Biostatistics, School of Public Health, Tehran University of Medical Sciences, Tehran, Iran; 5 Department of Clinical Epidemiology, School of Health and Safety, Shahid Beheshti University of Medical Sciences, Tehran, Iran; 6 Orbital and Oculoplastics Service, Farabi Eye Hospital, Tehran University of Medical Sciences, Tehran, Iran; 7 Department of Ophthalmology, Poostchi Ophthalmology Research Center, School of Medicine, Shiraz University of Medical Sciences, Shiraz, Iran

**Keywords:** Uveal melanoma, Radioactive plaque therapy, Enucleation, Survival

## Abstract

**Background:**

To assess the characteristics and outcomes of uveal melanoma management at a tertiary center in the Middle East.

**Methods:**

A study on 164 patients with uveal melanoma was conducted by reviewing the available medical records, ultrasound, and pathology report results. Age at diagnosis, tumor location and size, treatment mode, visual outcome, metastasis, mortality, and survival were studied.

**Results:**

The mean age of patients was 52.0 ± 15.0 years, and 52.5% were male. Choroidal melanoma was the most common uveal melanoma, followed by the ciliary body and iris melanoma. The mean thickness of tumors was 8.29 ± 3.29. The majority of patients (*n* = 111, 67.9%) were managed by brachytherapy with ruthenium-106 plaques. Enucleation was performed primarily in 46 (28%) patients and secondarily in nine (5.5%) patients. The sexual disparity was detected as the proximity of uveal melanoma to the fovea in males. For a 61-month mean follow-up period, mortality occurred in eight of our cases, six of which were due to metastasis. The most common site for distance metastasis was the liver (5/6), followed by the lung (1/6). The five-year and eight-year overall survival (OS) rate was 0.947%± 0.019. The 5-year survival rate reached zero in metastatic patients. OS was not statistically different depending on the age, tumor diameters, the primary treatment received, or the histopathologic findings (*p* > 0.50 for all).

**Conclusion:**

In this study, individuals diagnosed with UM exhibited an OS rate of around 94% at the five-year mark, which remained consistent up to eight years. Notably, the presence of distance metastasis emerged as the sole statistically significant factor influencing overall survival.

## Background

Uveal melanoma (UM) is the most common primary intraocular tumor, although it is rare [1]. Uveal melanomas differ from cutaneous melanomas in their etiology, biology, epidemiology, clinical course, and treatment options [2]. The most common site for UM is the choroid (90%), followed by the ciliary body (6%) and iris (4%) [3]. Several risk factors have been identified, including age, gender, race, skin color, oculodermal melanocytosis, cutaneous or ocular nevi, and exposure to sunlight and artificial ultraviolet light [4].

The incidence of UM has been reported at 5.1 cases per million people per year in the United States [5]. However, in parts of Africa and Asia, it has been reported as 0.2–0.3 cases per million people per year and as high as 9.2 in Ireland [6, 7]. A north-south gradient has been attributed to the protective role of ocular pigmentation, which is darker near the equator [8]. The median age at diagnosis is about 61–79 years, according to different reports that may vary by ethnicity [4, 9–11]. According to various studies, the mean age of onset in Asians ranges from 40 to 55, a decade or two earlier than in Western countries [1, 4, 12–14]. Men are more likely to be affected than women (1.3:1) [11]. 

Therapeutic modalities such as brachytherapy and proton beam irradiation, are the main conservative therapies, but enucleation remains the treatment of choice for larger tumors [3, 5]. The latest advances in therapy have resulted in a reduction in the number of primary enucleations performed [15]. 

In the study by Singh et al., the 5-year relative survival rate for UM was reported to be 77 − 84% [16]. Similarly, the 4-year overall survival rate reported by Baily et al. was 84% [7]. Almost half of UM cases eventually develop metastases within 30 years of treatment, and according to long-term follow-ups, the median survival time after metastasis is 6–24 months [5, 17, 18]. 

The presentation and outcomes of UM are varying in different parts of the world [13]. There are few reports regarding the prevalence, natural course, and outcome of UM disease in the Middle East, including Iran. Herein, we present findings from a study on clinical features, management, and outcomes of uveal melanoma in individuals from Iran, conducted at a tertiary referral center.

## Methods

This research was a retrospective chart review conducted by examining the medical records of patients with UM who were Managed at Farabi Eye Hospital in Tehran, Iran, from January 2008 to December 2020. Institutional review board approval was obtained from the Tehran University of Medical Sciences and adhered to the tenets of the Declaration of Helsinki. Since the present work is a retrospective study, informed consent was waived per our university policy.

All Iranian patients diagnosed with uveal melanoma (UM) and referred to the hospital were included in the study. The first visit, diagnosis, initial treatment plan, and patient follow-up were conducted in the ocular oncology service of Farabi Eye Hospital, providing us with sufficient mastery of the recorded information. All the patients have been followed for at least 3 years.

The diagnosis of uveal melanoma was based on clinical and ultrasonographic findings, all of which were confirmed by one expert specialist in ocular oncology (F.G and H.RE).

Each melanoma case was reviewed for the following variables: age, family history (FH), chief complaint (cc), gender, race, presenting intraocular pressure (IOP), number of tumors, size, location, color, presence of melanocytosis, distance to the optic nerve and fovea, extraocular extension, ultrasound findings, pathology results, management, complications, metastasis, death, and follow-up time. Additionally, specific tumor features (mushroom, dome, flat or diffuse) and the presence of drusen, halo, subretinal fluid, orange pigment, and pigment dispersion were noted. According to collaborative ocular melanoma study (COMS study), tumors were categorized as small (< 2.5 mm), medium (≥ 2.5 mm and ≤ 10 mm), and large (> 10 mm) based on tumor thickness [19]. 

Following the treatment protocol for UM patients, all of these patients were referred for medical monitoring for their cancer. They underwent blood tests, including liver function tests, as well as liver imaging (such as ultrasound and MRI) and chest X-ray, both at baseline and at specified intervals after treatment.

Treatment was based on tumor size, location, and visual potential; however, the patients’ preferences were an important factor. In Iran, the available treatment modalities for UM are brachytherapy, enucleation, and resection (partial lamellar sclerouvectomy in selected patients). Also, transpupillary thermotherapy (TTT) and photodynamic therapy (PDT) are adjunctive treatments that we often use in selected patients. As there is no proton-beam facility in Iran, we had to manage peripapillary UM tumors using other methods.

Brachytherapy with is the primary treatment for UM at Farabi Eye Hospital and was utilized for tumors up to 10 mm in thickness. In general, ^106^Ru plaques were employed to treat tumors up to 7 mm in thickness, while iodine-125 (^125^I) was used for tumors between 5 and 12 mm during a limited availability period [20]. In certain instances, brachytherapy was applied to address tumors exceeding 10 mm in thickness; For example, this approach was considered in cases involving the sole functioning eye or when patients declined enucleation and acknowledged the additional risks associated with prolonged eye radiation. As a general practice, enucleation was reserved for uveal melanomas measuring over 10 mm thickness or in the presence of other complications such as severe vitreous hemorrhage, iris neovascularization, and neovascular glaucoma (NVG).

Histological analysis was performed for all cases after enucleation. Fine needle aspiration and biopsy were performed for a few patients. Based on the pathology report, the tumors were categorized as spindle, epithelioid, or mixed cell type. Extra scleral extension (EOE), including optic nerve involvement, was also noticed in pathology reports. Molecular genetic testing was not evaluated for any patient.

The collected variables were analyzed using SPSS-version 24 software (IBM, Armonk, NY, USA). To describe quantitative data, statistical indices such as mean, standard deviation, and qualitative representations of ratios and statistical tables were used. For the evaluation of categorical variables, Chi-Squared test was used. In continuous variables, we used Kruskal-Wallis and Mann-Whitney tests for analyzing recorded dates. The Kaplan-Meier method was used to estimate survival times and the Log-rank test was used to compare differences in survival. A P-value less than 0.05 is considered significant.

## Results

Of the 164 patients, 86 (52.5%) were male and 78 (47.5%) were female. The baseline demographics and clinical characteristics of patients are summarized in Table 1. The mean age of patients was 52.0 ± 15.0 years. In most case (*n* = 145, 88.4%), the tumors were in the choroid, followed by the ciliary body (*n* = 17, 10.4%) and iris (*n* = 2, 1.2%). The mean thickness and maximum diameter of all tumors were 8.29 ± 3.29 mm and 14.20 ± 3.62 mm, respectively.

**Table 1:** The demographic data of the uveal melanoma patients

**Age in years (mean ± SD, range)**		52 ± 15	15–84	**Tumor Shape (N, %)**	**Mushroom**	71	43.30%
**Patient's gender (N, %)**	**Female**	78	47.60%		**Dome**	78	47.60%
	**Male**	86	52.40%		**Flat**	11	6.70%
					**Diffuse**	4	2.40%
**Vision (logMar, mean ± SD, range)**		1.13 ± 1.2	0–5	**Lesion Color (N, %)**	**Melanotic**	156	95.12%
**IOP (mean ± SD, range)**		14.72 ± 2.8	7–33		**Amelanotic**	7	4.26%
					**Mix**	1	0.60%
**Weight (Kg) of patient (mean ± SD, range)**		71 ± 13	38–107				
**Height (cm) of patient (mean ± SD, range)**		163 ± 13	50–192	**Pigment dispersion (N, %)**	**Yes**	47	28.70%
**Body Mass Index (mean ± SD, range)**		26 ± 4	17–43		**No**	117	71.30%
				**Iris Nevus (N, %)**	**Yes**	3	1.80%
**Smoking (N, %)**	**Yes**	52	31.70%		**No**	157	95.70%
	**No**	112	68.30%				
**Alcohol (N, %)**	**Yes**	24	14.60%	**Basal diameter (mean ± SD, range)**		14.20 ± 3.62	1.50–22.5
	**No**	140	85.40%	**Thickness of lesion (mean ± SD, range)**		8.29 ± 3.29	1.14–16.43
				**Distance to fovea (mm, mean ± SD, range)**		3 ± 4	0–16
**Duration of symptoms (Weeks, Mean ± SD, range)**		103.41 ± 103.76	85–1395	**Distance to optic disc (mm, mean ± SD, range)**		3 ± 4	0–17
				**Orange pigment (N, %)**	**Yes**	94	57.30%
**Symptoms (N, %)**	**Redness**	4	2.40%		**No**	70	42.70%
	**Pain**	15	9.10%	**Drusen (N, %)**	**Yes**	1	0.60%
	**Flashing**	16	9.80%		**No**	87	54.00%
	**Blurred Vision**	120	73.20%		**N/S**	73	45.30%
	**Floater**	7	4.30%	**Halo (N, %)**	**Yes**	1	0.60%
	**N/S**	2	1.20%		**No**	88	53.70%
					**N/S**	75	45.70%
**Type of disease (N, %)**	**CH Melanoma**	145	88.40%				
	**Ciliary body Melanoma**	17	10.40%				
				**Subretinal fluid (N, %)**	**Yes**	138	84.10%
	**Iris Melanoma**	2	1.20%		**No**	155	96.20%
				**Extraocular extension**	**Yes**	4	2.50%
**Laterality (N, %)**	**Left**	76	46.30%		**No**	160	97.5%
	**Right**	88	53.70%				
				**Treatment (N, %)**	**Brachytherapy I-125**	13	7.90%
**Quadrant Location (N, %)**	**Superior**	22	13.40%		**Brachytherapy** ^**106**^ **Ru**	98	57.5%
	**Nasal**	22	13.40%		**TTT**	4	2.40%
	**Inferior**	29	17.70%		**Enucleated**	46	28.00%
	**Temporal**	25	15.20%				
	**Peripapillary**	11	6.70%		**PDT**	3	1.80%
	**Inf-Temporal**	8	4.90%		**Brachy/enucleation/refused**	4	2.40%
	**Inf-Nasal**	14	8.50%				
	**Sup-Temporal**	11	6.70%				
	**Sup-Nasal**	11	6.70%				

^125^I: Iodide-125, IOP: Intraocular pressure, logMar: Logarithm of the Minimum Angle of Resolution, N: Number, N/S: Not stated, PDT: Photodynamic therapy, ^106^Ru: Ruthenium-106, SD: Standard Deviation, TTT: Transpupillary thermotherapy

Choroidal melanoma was presented as a dome-shaped mass in 78 patients (47.60%), mushroom-shaped in 71 patients (43.30%), flat lesion (thickness less than 3 mm) in 11 patients (6.70%), and diffuse type in four patients (2.4%). The lesion was pigmented in 156 patients (95.12%), amelanotic in seven patients (4.26%), and mixed in just one patient (0.60%). Subretinal fluid was present in 138 eyes (84.10%), and orange pigment was detected in 94 (57.30%) patients at the initial examination. Most of the patients (73.2%) experienced blurred vision, followed by flashing (10%) and pain (9.1%) (Table 1).

There was no significant difference between males and females regarding tumor thickness (*p* = 0.169), diameter (*p* = 0.956), or patient age (*p* = 0.342). Interestingly, UM in male patients was significantly closer to the fovea (*p* = 0.048) and was more likely to be present in superior quadrants (*p* = 0.029). Meanwhile, UM in females was more likely to present with inflammatory symptoms such as pain (*n* = 12 vs. *n* = 3) and redness (*n* = 4 vs. *n* = 0) while blurred vision was significantly more likely to be present in males (*n* = 69 vs. *n* = 51, *p* = 0.002). There were no significant differences between the two genders in terms of EOE (*n* = 2 vs. *n* = 1) or tumor pathology diversity (*p* = 0.435).

Regarding tumor thickness, 84 patients (51%) of patients presented with large melanoma (≥ 10 mm) on their first visit. The UM lesions were classified based on tumor thickness into small (*n* = 2), medium (*n* = 78), and large-sized tumors (*n* = 84). Large tumors were more likely in the temporal quadrants, while medium-sized tumors were more frequent in the superior quadrants (*p* = 0.007). Compared to small and medium-sized tumors, patients with large lesions were more symptomatic (*p* = 0.049), and more likely to have mushroom shaped tumors (*p* = 0.001). There was also a higher probability of detecting subretinal fluid in these patients (*p* = 0.001). Neither tumor thickness (*p* = 0.65) nor the tumor’s histopathologic type (*p* = 0.92) had a significant impact on the hazard of occurrence of EOE.

The majority of patients in the present study (*n* = 111, 67.9%) were managed by brachytherapy with ^106^Ru plaques (*n* = 98, 88.3%) and ^125^I plaques (*n* = 13, 11.7%). The mean age of patients who underwent brachytherapy was 50.90 ± 13.90 years and the mean tumor thickness and maximum diameter were 7.20 ± 2.65 mm and 13.54 ± 3.69 mm, respectively. Brachytherapy with ^106^Ru was performed for four patients who had tumor thicknesses of more than 10 mm. These patients refused enucleation but all these patients underwent secondary enucleation because of an uncontrolled disease. 25% of the brachytherapy patients underwent TTT laser as an adjunctive treatment. TTT was used as the only treatment method in four patients, and another three patients were treated with PDT and monitored for any signs of growth without any need for interventions. Periodic photographic documentation had shown no documented growth during at least five years of follow-up. It is important to note that it was not routine practice at our center to perform a biopsy for cytology or cytogenetic studies on patients undergoing brachytherapy treatment. The histology samples were from patients who underwent primary or secondary enucleation.

Primary enucleation was performed as the first line of treatment for 46 (28%) patients, whose mean age was 56.17 ± 15.45 years. The mean tumor thickness and maximum diameter in enucleated patients were 11.49 ± 2.49 mm and 16.4 ± 2.07 mm, respectively. The histopathology diversity for primary enucleated globes was: spindle cells type (*n* = 27, 59%), epithelioid cells (*n* = 13, 28%), and mixed cells (*n* = 6, 13%).

The mean follow-up period was 60.45 ± 22.21 and 60.56 ± 29.86 months for the brachytherapy and enucleation groups, respectively (*p* = 0.65). Secondary enucleation was performed in nine patients who failed conservative treatments (five due to local recurrence, two due to complications such as dense vitreous hemorrhage and glaucoma, and two due to the patient’s desire). The pathology reports for secondary enucleated globes were spindle type in four patients, epithelioid in 3 patients, and mixed type in two patients. Three patients (1.8%) experienced EOE. None of them had a local recurrence or developed metastatic disease.

During this follow-up period, 8 cases (6 males vs. 2 females) experienced mortality, with 6 of them attributed to metastasis. The characteristics of these cases are summarized in Table 2.

**Table 2:** The characteristics of deceased patients with uveal melanoma

	Age at presentation	Gender	Symptoms	Initial BCVA	Final BCVA	Treatment	Time to death (months)	Cause of death	Presence of metastasis	Location of metastasis	Diameter (mm)	Thickness (mm)	Location of the lesion	Extraocular extension	Pathology	Pigmentation	Adjuvant therapy	Laterality
1	61	Male	Visual loss	1/10	-	Enucleation	21	Metastasis	Yes	Liver	18.96	11.19	Choroid	No	Mixed cell	Pigmented	Interferon alpha 2b	OS
2	70	Male	Visual loss	LP	-	Brachytherapy then enucleation	16	Metastasis	Yes	Liver	16.72	12.91	Ciliary body	No	Spindle B	Pigmented	-	OD
3	37	Female	Pain - swelling	NLP	-	Enucleation	38	Metastasis	Yes	Liver	13	8.2	Choroid adjacent to disc	No	Mixed (necrotic)	Pigmented	-	OD
4	60	Male	Visual loss	4/10	CF 1 m	Brachytherapy	35	Metastasis	Yes	Lung	9.03	3.21	Choroid	No	-	Pigmented	-	OD
5	21	Male	Metamorphopsia	3/10	CF 1 m	Brachytherapy	58	Metastasis	Yes	Liver	20.58	11.77	Choroid	No	-	Pigmented	TTT laser	OD
6	59	Female	Visual loss	3/10	LP	Brachytherapy	34	-	No	-	11.17	4.27	Choroid adjacent to disc	No	-	Pigmented	-	OS
7	82	Male	Visual loss	LP	-	Enucleation	26	-	No	-	14.32	16.87	Choroid	No	-	Pigmented	-	OS
8	71	Male	Visual loss	CF2m	CF1m	Brachytherapy	44	Metastasis	Yes	Liver	15	5.7	Choroid	No	-	Pigmented	-	OD

BCVA: Best corrected visual acuity, CF: Count finger, LP: Light perception, NLP: No Light perception

Of patients with distance metastasis, three had undergone brachytherapy, two had undergone enucleation, and one had undergone enucleation after brachytherapy. The most common site for distance metastasis was the liver (5/6) followed by the lung (1/6). The mean tumor thickness at presentation in patients who did not have metastatic disease at the last follow-up was 7.84 mm; by comparison, it was 8.30 mm in those who did have metastatic disease (*p* = 0.9). All patients with metastasis died within five years of diagnosis. Unfortunately, records giving the exact times between the first exam and the detection of metastasis were not available.

**Fig. 1:**
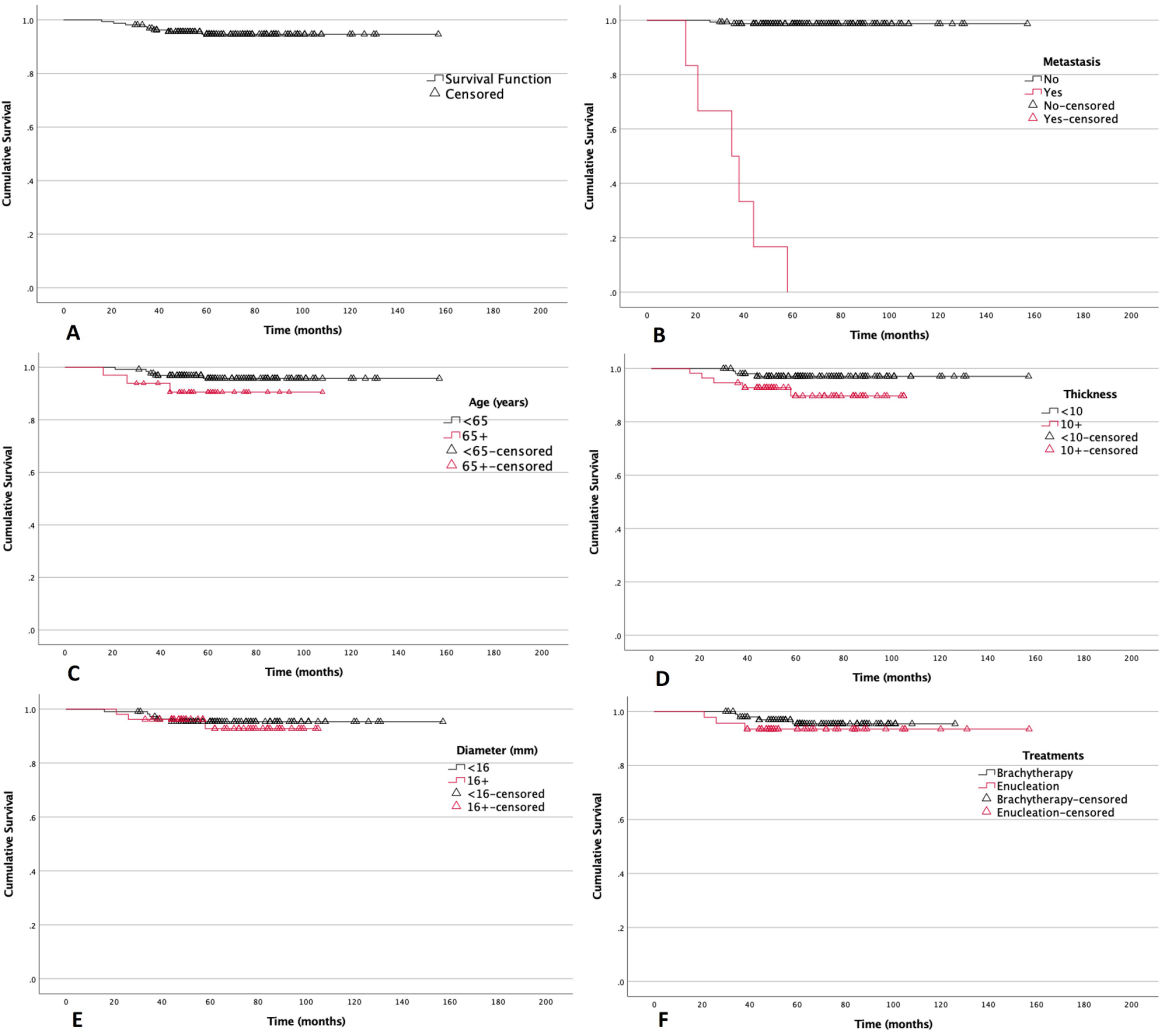
Overall survival analysis. (**A**): Overall survival. Time: Time following primary treatment, months. (**B**): Overall survival by distance metastasis. Time: Time following primary treatment, months. (**C**): Overall survival by age. Time: Time following primary treatment, months. (**D**): Overall survival by tumor thickness. Time: Time following primary treatment, months. (**E**): Overall survival by tumor diameter. Time: Time following primary treatment, months. (**F**): Overall survival by primary treatment (Brachytherapy vs. Enucleation). Time: Time following primary treatment, months

The overall survival (OS) curves are shown in Fig. 1A. The four and five-year OS rates (mean ± standard error) were 0.956 ± 0.016 and 0.947 ± 0.019,

respectively, based on 146 and 100 cases within the specified time frames.

The seven- and eight-year survival rates remained at 0.947 ± 0.019, in 42 and 20 cases who were followed up with at the time of analysis (Fig. 1A).

Distance metastasis was the only statistically significant factor affecting the OS (*p* < 0.001). The four-year OS rates were 0.987 ± 0.009 and 0.167 ± 0.152 in patients without and with distance metastasis, respectively. The five-year survival reached zero in metastatic patients, while in non-metastasis patients, the survival remained high at eight years (Fig. 1B).

OS was not statistically different depending on the age (*p* = 0.177), tumor thickness (*p* = 0.080), tumor diameter (*p* = 0.722), lesion pigmentation(*p* = 0.651) the primary treatment received (*p* = 0.478), and the histopathologic finding (*p* = 0.87).

The four-year OS rates were 0.969 ± 0.015 and 0.906 ± 0.052, for those aged 12–65 and 65 + years, respectively (Fig. 1C). The four-year OS rates were 0.971 ± 0.017 and 0.928 ± 0.035 for those with tumors measuring less than or equal to 10 mm and those measuring more than 10 mm, correspondingly. (Fig. 1D). The four-year OS rates for individuals with tumors diameter less than or equal to 16 mm and those measuring more than 16 mm were 0.953 ± 0.020 and 0.962 ± 0.026, respectively (Fig. 1E). The four-year overall survival rates were 0.969 ± 0.018 for those treated with brachytherapy and 0.935 ± 0.036 for those treated with enucleation. (Fig. 1F)

## Discussion

In this retrospective study on patients with uveal melanoma over the last 10 years, the mean age of patients was 52.0 years, and the mean tumor thickness and maximum diameter were 8.29 and 14.20 mm, respectively. Male patients had higher superior choroid UM lesions and more foveal lesions than females. The five-year and eight-year mortality rate is low in this study. All the metastasis in the cases occurred within the first five years after the diagnosis and all the cases deceased in the first five years after diagnosis.

Race appears to have matter in the occurrence of UM [14, 21], even in the United states, Cormier et al. reviewed the Surveillance, Epidemiology, and End Results (SEER) data from 1992 to 2002 for primary cutaneous melanoma in all races [22]. In that cohort, they identified 48,143 Caucasians (97%), 932 Hispanics (2%), 251 African Americans (0.1%), 52 American Indians (0.1%), and 294 Asians or Pacific Islanders (1%). They observed that non-Caucasians were two to four times more likely to present with advanced stage IV cutaneous melanoma, compared with Caucasians. Furthermore, the five-year survival was 90% for Caucasians and 72–81% for non-Caucasians. After adjustment for age, sex, and region, they found that non-Caucasians demonstrated a two- to threefold greater risk for melanoma-specific mortality compared with Caucasians.

Hu et al. noted that the lower incidence of cutaneous melanoma in non-Caucasians was ascribed to the protective effects of skin and eye pigmentation, but acknowledged that Hispanics, like Caucasians, were experiencing an increase in incidence, and recommended sun-protection measures for all races [23]. 

Hu et al. used the Surveillance, Epidemiology, and End Results (SEER) database to study the incidence of UM in different races from 1992 to 2000. The age-adjusted incidence of UM (per million people) was 0.3 for Blacks, 0.4 for Asians, 1.7 for Hispanics, and 6.0 for non-Hispanic Whites [24]. Except for Blacks versus Asians, the differences in incidence rates were statistically significant. When compared to Black patients, Asians had a relative risk of melanoma development of 1.2, Hispanics had a risk of 5.4, and non-Hispanic Whites had a risk of 19.2 [24]. 

The prevalence of UM varies by geographic location. The incidence has been reported to be between 2 and 8 per million for the European population. [8] The study by Virgili et al. [8] described geographic variability in the incidence rate from the north to south of Europe. Baily et al. showed that the mean age-adjusted incidence of UM in Ireland was 9.5 per million people [7]. Singh et al. collated prior studies on the incidence of UM worldwide from 1961 to 2001 and found that incidence rates ranged from 0.3 per million in Japan to between 9.0 and 10.4 per million in Norway, Sweden, East Germany, and Ohio (USA) [16, 18]. 

While the information available in the current study cannot estimate the prevalence of UM in Iran, given the racial similarities (darker skin pigmentation) and ambient geographic similarities (light exposure) between Iranians and those living in southern Europe such as Spain or Italy, a similar prevalence appears to be warranted for Iran.

The mean age at diagnosis of UM in this study was 52 years, which is lower than reported in the United States and Europe [3, 7, 14]. Shields et al. reported a mean age of 58 years for 8,033 patients with UM in the United States [3]. In Ireland, Baily et al. documented a mean age of 61.7 years for 253 patients with UM [7]. Compared with these, the mean age of patients in our study is lower and is close to that reported in studies from other Middle Eastern countries such as Saudi Arabia (50 years) and Jordan (46 years) [25, 26]. According to the other studies [14], a comparative cohort study of Chinese and Asian American UM patients revealed that Asian patients were diagnosed at a younger age (47.3 ± 12.5 years vs. 59.7 ± 14.8 years) [21, 27]. 

Kuo et al. [28] evaluated the findings of 65 Chinese patients with UM in 1982 and found that they presented at a younger age compared to Caucasians from the United States.

Biswas and colleagues examined 103 Asian Indian eyes with UM and compared them to other ethnic groups. They observed that UM in Asian Indians is more common in younger individuals (mean age 46 years) and has a larger diameter (mean basal diameter 13 mm) than in Caucasians [29]. 

Shield et al. [14] found that Caucasians presented with uveal melanoma at an older age (58 years) compared to non-Caucasians (44–52 years). Despite most Iranian being identified as Caucasian [30], the incidence age of uveal melanoma in this study was more similar to non-Caucasians in Western countries.

In this study, 52.5% of the subjects were male. However, except for tumor location and distance from the macula and metastasis, we found no statistical difference in the studied parameters between men and women. Similar to the present study, Damato et al. [31] described differences in tumor location with a tendency toward the thicker and posterior location of tumors in men. Another study showed greater involvement of the ciliary body and iris in women [32]. Concordant with our study, this study also demonstrated more posterior tumors and an increased rate of metastasis and melanoma-related mortality in men [32]. Similarly, Rietschel et al. and Zloto et al. noticed that the male gender correlated independently with a significantly higher risk of melanoma-related mortality [32, 33]. Kujala et al. found no gender differences in melanoma-related mortality [34]. 

In Baily et al.’s study, UM location was choroidal in 82%, ciliary body region in 11%, and iris in 7% of cases [7]. In the study by Shields et al., the choroid was the most common location of UM (90%), followed by the ciliary body (6%) and the iris (4%) in the American population [3]. The percentages of cases presenting these tumor sites in the present study were 88.4%, 10.4%, and 1.2%, respectively.

Clinical factors associated with the prognosis and death of all American patients with UM in multivariate analysis included higher age, more tumor thickness and basal diameter, diffuse tumor morphology, ciliary body location, higher pigmented tumors, the presence of subretinal fluid, extraocular extension, and the presence of intraocular hemorrhage [3]. The multivariate factor for metastasis in Hispanics was increasing tumor basal dimension (*p* = 0.039). There were no significant factors for metastasis in Asians or African Americans [3]. 

Tumor diameter (especially thickness) at diagnosis was believed to be the most important clinical prognostic factor related to prognosis and patients’ survival [35, 36]. A study by Kestel et al. showed that the overall five-year survival rates of patients decreased from 66.7% for smaller UM tumors (≤ 9 mm) to 28.6% for larger tumors (9.1–15 mm) [37]. A study by Shields et al. on 8033 μm cases demonstrated that metastasis at five and 10 years was 6% and 12%, for small melanoma (0–3.0 mm thickness), 14% and 26%, for medium-sized tumors (3.1–8.0 mm), and 35% and 49% for large UM tumors (> 8.0 mm) [3]. In the present study, four of six patients with systemic metastasis (66.6%) had large UM.

In the present study, the mean tumor thickness and diameter at diagnosis were 8.29 ± 3.29 mm and 14.20 ± 3.62 mm, respectively; these values are larger than indicated in reports from European and American patients [3, 5, 8]. The mean tumor thickness and diameter reported in Ireland by Baily et al. were 6.5 ± 3.8 mm and 12.5 ± 3.6 mm, respectively [7]. In another study from the USA on SEER database, the mean tumor thickness and diameter were 4.9 ± 3.01 mm and 11.3 ± 8.27 mm, respectively [38]. Also, Shields et al. reported a thickness and basal diameter of 5.5 mm (4.5, 1–24) and 11 mm (11, 1–33), respectively. Similar to the present study, the tumor thickness (7.1 ± 3.28 mm) and basal diameter (12.0 ± 3.54 mm) were significantly high in a study on Chinese patients [21]. 

Apparently, tumors are larger at the time of diagnosis in Iran and other Asian countries compared to Western countries [21]. We hypothesize that awareness of UM signs and symptoms among general and subspecialty ophthalmologists and improving general population education can lead to earlier diagnosis and improved prognosis of UM during routine eye examinations. According to this hypothesis, a high percentage of patients in this study had mushroom-shaped tumors (43.4%); by comparison, approximately 19% of patients had such tumors in the study by Shields et al. [3].

In the current study, the lesions were pigmented in 155 patients (95.1%) and amelanotic in only seven patients (4.3%). Previous studies have shown that about 15–25% of choroidal melanomas in Western countries are amelanotic melanomas which are thought to arise from an amelanotic nevus [3, 39]. Zewar et al. reported an amelanotic choroidal melanoma rate of 7% in Jordan, and Shields reported a rate of 16% in African Americans and 14% in Asian Americans. The presence of pigmentation in the tumors has been associated with the growth of UM and has been shown to have a poorer prognosis than amelanotic ones [39]. Our study showed no correlation between OS and lesion pigmentation.

With the introduction of brachytherapy as an effective treatment method for UM in the early 70s, the number of eyes undergoing enucleation as primary therapy gradually decreased. Multiple radiotherapy techniques (plaque brachytherapy, proton beam radiation, etc.) have been introduced. Radiotherapy, which only accounted for only 10% of cases in the early 80s, reached 70% in 2014 [36]. Brachytherapy was performed in 73% of Chinese patients and almost two-thirds of UM patients in Ireland [7, 21]. In the present study, 65% of all patients were treated with brachytherapy, aligning with findings in other studies. Alsuhaibani et al. reported an 83% enucleation rate. The high rate of enucleation in our region might be attributed to the more prevalence diagnosis of large-sized tumors at the initial presentation [26]. Based on up to 12 years of follow-up, the COMS data suggest that mortality rates among patients treated with eye-conserving I_125_ brachytherapy were comparable to mortality rates among patients treated with enucleation. This included deaths from all causes as well as tumor-specific mortality [40]. 

Despite evolving trends towards eye-sparing treatment, the 5-year relative survival rate has remained unchanged over the past 40 years, and approximately 50% of uveal melanoma patients will develop metastatic disease within 30 years of diagnosis [3]. The COMS found cumulative metastasis rates of 25% and 34% after five and 10 years, respectively, with 80% of metastatic patients dying within one year and 92% dying within two years of being diagnosed with metastases [41]. In a review by Singh et al., the 5-year relative survival rate (81.6%) remained stable during the observation period from 1973 to 2008 [5]. Using SEER data, Bishop et al. reported a 5-year survival rate of 78.4% for 7069 uveal melanoma cases. [35]. Another study, on 1500 μm cases in China reported a 5-year survival rate of 84.0% for their patients [21]. In a study on 253 Irish patients by Baily et al. the overall four-year survival was 84% and the OS was significantly lower in patients who had undergone enucleation as well as patients over 65 years old [7]. The overall five-year survival rate for patients in our study was 94.7%. Although the pattern of treatment was the same as the Irish population, we did not observe any significant difference in patients’ survival between the treatment protocols and age. It shows that the better survival in patients who had undergone brachytherapy in the Irish study was not a result of the superiority of radiation over enucleation but rather due to other factors like the patient selection at presentation, race, or ethnicity. In a Jordanian study about 96% of patients were alive after a mean 2-year follow-up [25]. In another study in Saudi Arabia, metastasis was detected in 5% of patients after 5 years from the initial treatment of medium and large-sized UMs. Due to the very short follow-up period for more than half of the patients, they could not provide survival outcome among their patients population [26]. Though, Shields et al. found no statistical difference in metastasis or death when comparing the Caucasian population to the Hispanic, Asian, or African American populations [3]. 

The most common histopathological finding in primary enucleated globes was spindle cells type (59%), with epithelioid cells following at 28%. A similar pattern was reported by Luo et al. based on a Chinese database [42]. Notably, the histopathological analyses of five cases characterized as spindle cell type revealed spindle cells type A as the predominant cell population. While these enucleated eyes displayed a mixture of spindle A and spindle B cells, spindle A was predominantly observed. According to COMS report number 6, there are distinctions between spindle A cells in choroidal melanoma and spindle cell nevus. Unlike spindle cell nevus, which features abundant cytoplasm with a small nucleolus, spindle A cells are typically smaller, with sparse cytoplasm and a plumper nucleus [43]. Recently, the American Joint Committee of Cancer (AJCC) classification identifies three cell types: (i) spindle cell (typically comprising a mixture of spindle A and spindle B cells) (ii) epithelioid cell, and (iii) mixed cell type. According to this classification, spindle cell melanoma consists of spindle cells constituting ≥ 90% of the tumor, while epithelioid cell melanoma comprises epithelioid cells making up ≥ 90% of the tumor. All other tumors fall under the category of mixed cell melanoma. [44]. Research indicates that, compared to spindle cell melanomas, epithelioid cell melanomas, followed by mixed cell melanomas, exhibit a poorer prognosis [43]. 

It is also worth noting that our study is based on tertiary referral center patients’ data and cannot be generalized to the entire country. It could be postulated that some possible cultural, environmental, socioeconomic factors, and other undetermined factors may affect this disparity in the findings.

This is the first study to describe the clinical characteristics and outcomes of UM since brachytherapy began being used as an effective option for the treatment of UM at our center. The relatively small sample size is a limitation of this study. Moreover, Due to the constrained duration of our follow-up period, we regret to acknowledge that our study lacks the capacity to provide comprehensive insights into 10-year survival rates. This limitation arises from the inherent challenge of capturing long-term outcomes within the specified timeframe of our research. Consequently, our findings may not fully capture the nuances and trends that may emerge over a more extended period, specifically in the context of assessing 10-year survival rates. Another shortcoming of this research is that FANB, cytology, and chromosomal testing of samples could not be performed. Regardless of these limitations, our data offers considerable insight into the clinical features, treatment modalities, and survival of Iranian patients with UM.

## Conclusion

In this study, the average age at the time of uveal melanoma (UM) diagnosis was 52 years. The mean dimensions of the tumor at the point of diagnosis were 8.29 ± 3.29 mm in thickness and 14.20 ± 3.62 mm in diameter. The OS rate of these patients after 5 years was 94.7%, and this rate remained stable at 8 years of follow-up. Notably, this survival was not dependent on age, tumor thickness, tumor diameter, the primary treatment received, and the histopathologic finding. Distance metastasis emerged as the only statistically significant factor affecting OS.

## Data Availability

The datasets used in the current study are available upon reasonable request.

## Abbreviations

UM: Uveal melanoma
FH: Family history
cc: Chief complaint
IOP: Intraocular pressure
COMS study: Collaborative ocular melanoma study
TTT: Trans Pupillary Thermotherapy
PDT: Photodynamic Therapy
^106^Ru: Ruthenium-106
^125^I: Iodine-125
NVG: Neovascular glaucoma
FNAB: Fine needle aspiration and biopsy
EOE: Extra scleral extension
OS: Overall survival
